# BACH1 Promotes Temozolomide Resistance in Glioblastoma through Antagonizing the Function of p53

**DOI:** 10.1038/srep39743

**Published:** 2016-12-21

**Authors:** Er Nie, Xin Jin, Weining Wu, Tianfu Yu, Xu Zhou, Tongle Zhi, Zhumei Shi, Junxia Zhang, Ning Liu, Yongping You

**Affiliations:** 1 Department of Neurosurgery, the First Affiliated Hospital of Nanjing Medical University, Nanjing 210029, China; 2 State Key lab of Reproductive Medicine, Department of Pathology, Collaborative Innovation Center for Cancer Personalized Medicine, Cancer Center, Nanjing Medical University, Nanjing 210029, China; 3 Chinese Glioma Cooperative Group (CGCG), Nanjing, China.

## Abstract

The acquisition of drug resistance is a persistent clinical problem limiting the successful treatment of glioblastoma (GBM). However, the molecular mechanisms by which initially chemoresponsive tumors develop therapeutic resistance remain poorly understood. In this study, we report that BACH1, a heme-binding protein that participates in transcriptional repression or activation, was significantly upregulated in glioblastoma tissues. Overexpression of BACH1 in GBM cells conferred resistance to temozolomide, whereas its inhibition markedly sensitized resistant cells to temozolomide *in vitro* and *in vivo*. Further investigation revealed that BACH1 activation significantly enhanced the expression of MGMT, and depletion of p53 disrupted the effects of BACH1 on MGMT and temozolomide resistance. P53 sequesters SP1 to prevent its binding to the MGMT promoter region and thus inhibits MGMT expression. Moreover, BACH1 overexpression impaired the association between p53 and SP1 via competitive binding p53, and antagonized the impact of p53 on MGMT expression. Finally, we found that BACH1 low expression correlated with better prognosis in GBM patients undergoing temozolomide therapy, especially in patients with wild-type TP53. Collectively, our findings identify a potential mechanism by which wild-type TP53 GBM cells develop resistance to temozolomide and suggest that targeting this pathway may be beneficial for overcoming resistance.

The most common primary adult human brain tumors are gliomas, with grade IV glioblastoma multiforme (GBM) astrocytoma being most common and malignant^1^,^2^,^3^. The median survival of GBM patients is only approximately 12–16 months^1^. The standard of care for glioblastoma is composed of maximal surgical resection followed by radiotherapy with concomitant and adjuvant chemotherapy^4^,^5^. Temozolomide (TMZ) is a monofunctional alkylating agent currently used as the first-line chemotherapeutic agent against newly diagnosed glioblastoma and is also a drug of choice for recurrent disease^6^,^7^. Sensitivity of tumor cells to TMZ is therefore key to successful management of this intractable disease. Unfortunately, glioblastoma cells often exhibit resistance against this alkylating agent. Multiple independent DNA repair mechanisms that normally maintain genomic integrity can promote drug resistance and cancer cell survival by removing alkyl groups induced by chemotherapy. Among possible mechanisms, O^6^-methylguanine DNA methyltransferase (MGMT) expression has been well documented as the clinically most relevant mechanism of resistance against TMZ-based glioblastoma therapies^8^. MGMT is a repair enzyme that rapidly removes the methyl group attached by TMZ at the O^6^ position of the guanine residue and as such could theoretically counteract the antitumor effect of TMZ. Indeed, accumulating evidence from correlative observations as well as from functional analyses, in either clinical settings or *in vitro* studies^8^,^9^,^10^,^11^,^12^, now suggests that this is actually the case with glioblastoma, underscoring the absolute necessity for developing novel methods to inactivate MGMT in tumor cells to overcome TMZ resistance. Understanding the molecular mechanism involved in the regulation of MGMT expression/function is vital for identification of therapeutic targets; however, signaling pathways controlling MGMT in glioblastoma cells remain poorly characterized.

The transcriptional repressor BTB and CNC homology 1 (BACH1) is a heme-binding protein belonging to the cap′n′collar type of basic leucine zipper factors and constitutes a major link between the cellular heme level, the redox state, and the transcriptional response. BACH1 was identified as a key player in the physiological regulation of oxidative stress, where it acts as a repressor of its main target hemoxygenas-1^13^. Recently, growing evidence revealed BACH1 in the case of cancer progression. Liang *et al*.^14^ revealed that ectopic expression of BACH1 is involved in enhancement malignancy of breast cancer cells while knockdown considerably suppresses these processes. BACH1 transcriptionally adjust several involved genes in the osteolytic metastasis of breast cancer, and more significantly, it promotes the invasiveness and metastasis of breast cancer cells^15^. Therefore, BACH1 may be a significant target for effective therapeutic intervention in tumor metastasis development.

However, the role of dysregulated BACH1 in GBM biology remains unknown. In this study, we show that, in wild-type p53 glioblastoma cells, BACH1 dictates MGMT expression and TMZ resistance via antagonizing the function of p53 and may be leveraged as a novel potential therapeutic target for GBMs.

## Results

### BACH1 overexpression confers resistance to TMZ

**Figure 1:**
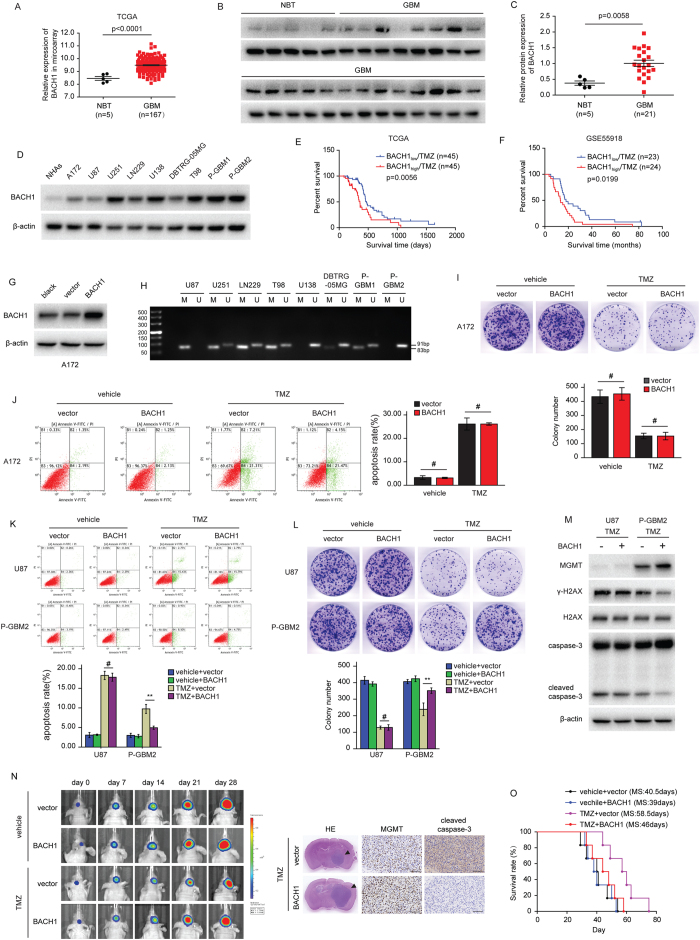
BACH1 overexpression confers resistance to TMZ. (**A**) Levels of BACH1 were analyzed in GBM and NBT of TCGA database. (**B**) The protein level of BACH1 in NBT (n = 5) and GBM (n = 21) tissues were examined by WB. (**C**) Expression of BACH1 protein was analyzed in NHAs and nine GBM cells. (**D**,**E**) Kaplan–Meier curves showing the overall survival of patients with high or low expression of BACH1 in GBM patients receiving TMZ therapy using the TCGA database and GSE55918 database. (**G**) Western blot analysis of BACH1 expression in A172 cells transfected with BACH1 or vector control, respectively. (**H**) MSP assay for methylation status of the MGMT promoter in GBM cells. PCR products in the M lanes and U lanes indicate methylated and unmethylated status of the MGMT promoter, respectively. (**I**,**L**) Colony formation assays were done with GBM cells infected with BACH1 or vector in the presence of TMZ (200 μM). (**J**,**K**) BACH1 overexpression GBM cells were exposed to 200 μM TMZ for 48 hours and the apoptosis was measured by flow cytometry. (**M**) Western blot analysis of MGMT, γ-H2AX, H2AX, caspase-3 and cleaved caspase-3 expression in GBM cells transfected with BACH1 or vector in the presence of TMZ (200 μM). (**N**) representative pseudocolor bioluminescence images of intracranial xenografts bearing BACH1-overexpressing P-GBM2 or vector control cells in the absence or presence of TMZ on the days as indicated. representative H&E staining for tumor cytostructure. IHC analysis of MGMT and cleaved caspase-3 expression in intracranial xenografts. (**O**) survival curve of BACH1-overexpressing P-GBM2 or vector control cells-derived intracranial xenografts treated with TMZ. GBM: Glioblastoma multiforme. NBT: nontumor brain tissue. P-GBM2: primary GBM2. NHAs: normal human astrocytes. Student’s t-tests and One-way ANOVA were performed. Data are presented as mean ± SEM (**P < 0.01, ^#^*P* > 0.05), scale bar = 100 μm.

The TCGA database of glioma showed that BACH1 levels were higher in GBM than nontumor brain tissue (NBT) (Fig. 1A), and this result was verified by the data from clinical samples (Fig. 1B,C). Next, we analyzed the levels of BACH1 in normal human astrocytes (NHAs) and nine GBM cell lines (A172, U87, U251, LN229, U138, DBTRG-05MG, T98, primary GBM1, and primary GBM2) using Western blot analysis. Almost all of them were found to highly express BACH1 in comparison with NHAs (Fig. 1D). Further analysis of BACH1 expression using the TCGA database and GSE55918 database revealed that high BACH1 expression correlated with poor survival in patients with GBM receiving TMZ therapy (Fig. 1E,F). These findings suggest a possible correlation between BACH1 expression and TMZ resistance.

To determine whether BACH1 is associated with TMZ resistance, we overexpressed BACH1 in A172 cells (TMZ sensitive cells lacking MGMT expression; Supplementary Fig. 1A,B), U87 cells (MGMT promoter methylation) and P-GBM2 (primary GBM2) cells (MGMT promoter hypomethylation) (Fig. 1G,H). As shown in Fig. 1I–L, TMZ inhibited cell viability and induced cell apoptosis in the three GBM cell lines. However, overexpressing BACH1 only rescued these change in P-GBM2 cells, but not in A172 and U87 cells. After TMZ treatment, P-GBM2 cells expressing BACH1 had a significant increase in the expression of MGMT and decrease in the levels of phosphorylated histone H2AX (γ-H2AX) and cleaved caspase-3 compared with vector cells (Fig. 1M). Yet BACH1 overexpression did not affect the levels of MGMT, γ-H2AX and cleaved caspase-3 in U87 cells (Fig. 1M). These results indicate that BACH1 overexpression protects GBM cells against TMZ-induced growth inhibition and apoptosis through MGMT.

To examine whether BACH1 confers resistance to TMZ *in vivo*, we intracranially injected P-GBM2 cells expressing BACH1 expression construct or vector control into immunocompromised mice. After confirmation of GBM engraftment, mice were treated with TMZ (66 mg/kg/day) or placebo for 5 days per week for three cycles. Mice injected with P-GBM2 cells expressing vector control had a significant decrease in tumor volume when treated with TMZ, whereas tumor growth of TMZ treated xenografts overexpressing BACH1 was almost not affected (Fig. 1N). And xenografts overexpressing BACH1 had significantly increased levels of MGMT and decreased levels of cleaved caspase-3 upon TMZ treatment in comparison with vector control xenografts. The median survival for TMZ plus vector-treated groups was 58.5 days, whereas the median survival for TMZ plus BACH1-treated groups was 46 days (n = 6/group, *P* < 0.01; Fig. 1O). Together, these findings show that BACH1 overexpression contributes to development of acquired resistance to TMZ *in vitro* and *in vivo*.

### BACH1 depletion sensitizes GBM cells to TMZ

**Figure 2:**
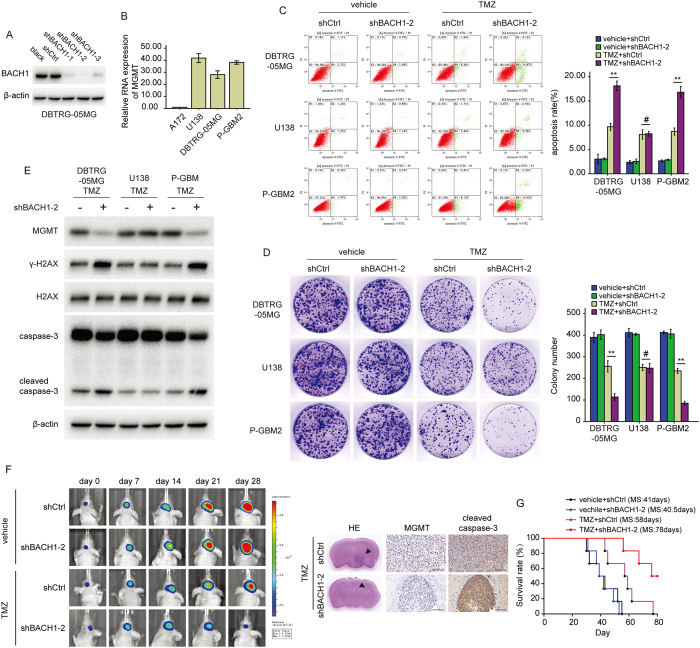
BACH1 depletion sensitizes GBM cells to TMZ. (**A**) Western blot analysis of the level of BACH1 in DBTRG-05MG cells transfected with three independent luciferase-encoding BACH1 shRNA or control shRNA. (**B**) The RNA level of MGMT in A172, DBTRG-05MG, U138 and P-GBM2 cells. (**C**) BACH1-depleted GBM cells were exposed to 200 μM TMZ for 48 hours and the apoptosis was measured by flowcytometry. (**D**) colony formation assays were done with GBM cells infected with lenti-shBACH1-2 or lenti-shCtrl in the absence or presence of TMZ. (**E**) Western blot analysis of MGMT, γ-H2AX, H2AX, caspase-3 and cleaved caspase-3 expression in BACH1–depleted GBM cells or control cells in the presence of TMZ. (**F**) representative pseudocolor bioluminescence images of intracranial xenografts bearing BACH1–depleted P-GBM2 or control cells in the absence or presence of TMZ on the days as indicated. representative H&E staining for tumor cytostructure. IHC analysis of MGMT and cleaved caspase-3 expression in intracranial xenografts. (**G**) survival curve of BACH1–depleted P-GBM2 or control cells-derived intracranial xenografts treated with TMZ. Student’s t-tests and One-way ANOVA were performed. Data are presented as mean ± SEM (**P < 0.01, ^#^*P* > 0.05), scale bar = 100 μm.

If gain-of-BACH1 function could confer resistance to TMZ in GBM cells, we asked whether loss of BACH1 could cause sensitization. We infected GBM cells with three independent luciferase-encoding BACH1 shRNA (short hairpin RNA) or control shRNA. Altered expression of BACH1 in GBM cells was confirmed by western blot analysis (Fig. 2A). We chose shBACH1–2 for the following study according to its knockdown efficiency. And U138, DBTRG-05MG and P-GBM2 cells (TMZ-refractory cells with MGMT promoter hypomethylation; Fig. 1H and Supplementary Fig. 1A,B) expressing the MGMT gene transcripts were selected for further study (Fig. 2B). After TMZ treatment, BACH1-depleted DBTRG-05MG and P-GBM2 cells showed an significant increase in apoptosis (Fig. 2C) and a reduction in colony-forming ability compared with shCtrl cells (Fig. 2D). In addition, knockdown of BACH1 led to a significant decrease in MGMT and an increase in γ-H2AX and cleaved caspase-3 expression (Fig. 2E). However the growth, apoptosis and indicated proteins levels of U138 cells were not affected.

Next, 2.5 × 10^5^ luciferase-labeled BACH1-depleted P-GBM2 cells were intracranially injected into NOD/SCID mice. The tumor-bearing mice were treated with TMZ (66 mg/kg/day) or vehicle control for 5 days per week for three cycles. During 21-day treatment, the control xenografts showed tumor progression. In stark contrast, xenografts carrying BACH1-depleted P-GBM2 cells displayed a significant regression of tumor growth following TMZ treatment (Fig. 2F). Histologic analysis confirmed that all mice bearing tumors derived from BACH1-depleted P-GBM2 cells had a significant decrease in MGMT expression and increase in cleaved caspase-3 expression (Fig. 2F). These findings were further confirmed by the survival curves, in which TMZ-treated BACH1-depleted xenografts exhibited significantly increased survival as compared with TMZ-treated control xenografts (n = 6/group, *P* < 0.01; Fig. 2G). Together, these data suggest that inhibition of BACH1 sensitizes GBM cells to TMZ *in vitro* and *in vivo*.

### BACH1 mediates epigenetic regulation of MGMT and TMZ resistance through antagonizing p53

Methylation of MGMT promoter is found in 40% of cancer types such as glioma and colorectal cancer and in 25% of non-small cell lung carcinoma, lymphoma and head and neck carcinoma^16^. The expression of MGMT protein is significantly reduced in MGMT promoter-methylated cancer cells^16^. To investigate the influence of BACH1 on MGMT promoter methylation, a methylation-specific PCR (MSP) assay was performed in GBM cells. However, either inhibiting or ectopically expressing BACH1 did not influence the methylation of CpG islands in the MGMT promoter (Supplementary Fig. 2A–D).

**Figure 3:**
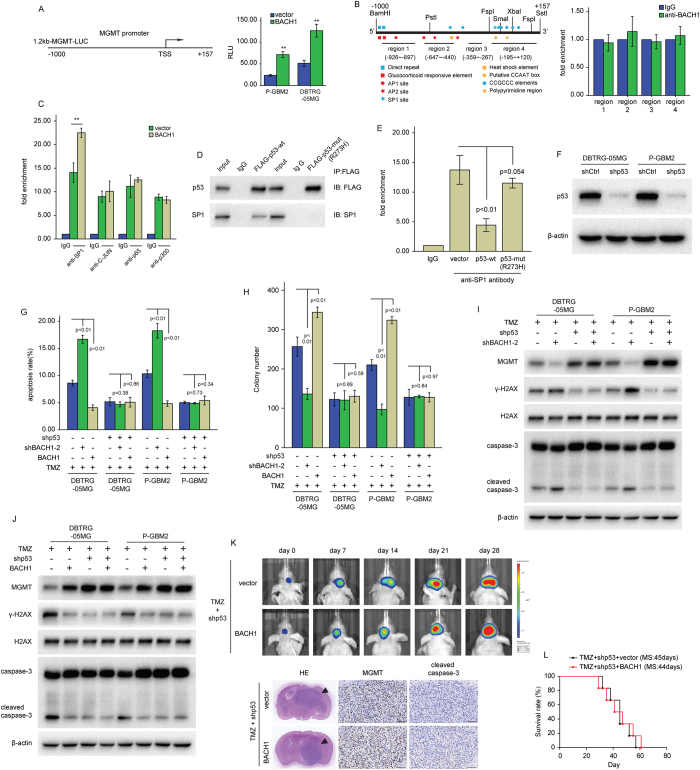
P53 knockdown abolished the effects of BACH1 on TMZ resistance *in vitro* and *in vivo*. (**A**) GBM cells were transfected with the reporter MGMT-Luc construct or with other plasmids as indicated. β-galactosidase constructs was included in each transfection to normalize transfection efficiency. A luciferase reporter assay was performed to measure MGMT promoter activity. TSS, transcription start site. (**B**) Schematic illustration of the promoter of the human MGMT gene and the region containing the primers for ChIP assay (left). ChIP assays were performed using anti-IgG or anti-BACH1 antibody. The eluted DNA was subjected to qRT-PCR with the specific primer set for the MGMT promoter region (right). (**C**) DBTRG-05MG cells transfected with BACH1 or vector was subjected to ChIP assays using the indicated antibodies. The eluted DNA was subjected to qRT-PCR with the specific primer set for the MGMT promoter region. (**D**) DBTRG-05MG cells transfected with FLAG-p53-wt or FLAG-p53-mut (R273H) was subjected to Co-IP analysis using the indicated antibodies. (**E**) DBTRG-05MG cells separately transfected with p53-wt, p53-mut (R273H), or empty vector was subjected to ChIP assays using anti-IgG or anti-SP1 antibody. The eluted DNA was subjected to qRT-PCR with the specific primer set for the MGMT promoter region. (**F**) Western blot analysis of p53 in GBM cells transfected with p53 shRNA or control shRNA. (**G**,**H**) GBM cells cotransfected with BACH1 or shBACH1-2, and shp53 were treated with TMZ(200 μM) for 48 hours. Flowcytometry and colony formation assays were used to measured cells apoptosis and proliferation. (**I**,**J**) GBM cells cotransfected with BACH1 or shBACH1-2, and shp53 were treated with TMZ(200 μM) for 48 hours. Western blot analysis of the indicated proteins expression. (**K**) Representative pseudocolour bioluminescence images of orthotopic tumors bearing vector control or ectopic expression BACH1 P-GBM2 cells stably expressing shp53 after treatment with TMZ on the days as indicated. IHC analysis of MGMT and cleaved caspase-3 expression in intracranial xenografts. (**L**) survival curve of vector control or ectopic expression BACH1 P-GBM2 cells stably expressing shp53-derived intracranial xenografts treated with TMZ. Student’s t-tests and One-way ANOVA were performed. Data are presented as mean ± SEM (**P < 0.01), scale bar = 100 μm.

As a heme-binding protein, BACH1 forms heterodimer through the BTB-POZ domain which binds to the DNA-binding complex and is involved in the regulation of the chromosomal structure, which turns out the transcriptional repression or activation^17^. To further investigate the mechanism by which BACH1 regulated MGMT expression, we generated a luciferase reporter construct containing ~1.2 kb of the distant MGMT promoter (Fig. 3A, left). The 1.2 kb MGMT promoter activity was decreased in BACH1-knockdown and increased in BACH1-overexpressing DBTRG-05MG cells (Fig. 3A, right), indicating that BACH1 up-regulated MGMT expression at the transcriptional level via the activity of the MGMT distal promoter. However, as shown by Chromatin immunoprecipitation (ChIP) assays, BACH1 could not occupy the promoter of MGMT (Fig. 3B). Different transcription factors have been found to activate the transcription of MGMT gene, including SP1^18^, p65^19^, p-300^20^ and AP-1^21^. Therefore, we next investigated SP1, p65, AP-1, and p-300 in the MGMT promoter region using ChIP followed by qRT-PCR. Only SP1 was increased at the MGMT promoter region after transfection with the BACH1 expression construct (Fig. 3C). Bocangel *et al*. reported that wild-type p53 sequesters SP1 to prevent its binding to the cognate *cis* elements in the MGMT promoter and thus inhibits MGMT expression, but not the p53LT mutant (p53 mutant Leu22Gln Trp23Ser)^22^. Our findings indicate that inhibition of BACH1 sensitizes DBTRG-05MG and P-GBM2 cells to TMZ, but not U138 cells. U138 cells (p53 mutant R273H) has a nonfunctional p53^23^,^24^, while DBTRG-05MG and P-GBM2 cells have a wild-type p53. The codon R273H mutant in the DNA-binding domain of p53 showed a reduction in the CAT activities and resulted in clear loss of function of p53 for its transcription factor phenotype, binding to the hdm-2 protein, and binding to the Ad5 E1B 55-kD protein^25^. Wild-type p53 and some of its mutants are known to form heterocomplexes with SP1^26^. Next we investigate whether p53-mutant-R273H could interact with SP1. To test this, we performed co-immunoprecipitation analysis of p53 in extracts of DBTRG-05MG cells transfected with FLAG-p53-wt or FLAG-p53-mut-R273H. Western analysis of the immunoprecipitate with anti-FLAG antibody showed the presence of SP1 in the FLAG-p53-wt immunoprecipitate but not in the FLAG-p53-mut-R273H (Fig. 3D). And overexpression of wild-type p53 showed reduced SP1 binding, while the p53-mut-R273H had no significant effect (Fig. 3E).

To further detect the role of p53 in BACH1-modulated TMZ resistance, we knocked down the expression of p53 in DBTRG-05MG and P-GBM2 cells transduced with BACH1 or shBACH1–2 (Fig. 3F), and treated those cells with vehicle or TMZ. Knockdown of p53 observably rescued the cell apoptosis and the reduction of colony forming capacity induced by TMZ. And overexpression or downregulation of BACH1 did not affect the function of TMZ in GBM cells in the absence of p53 (Fig. 3G,H). Of note, knockdown of p53 abolished the shBACH1-mediated attenuation of MGMT and the enhancement effect of BACH1 on MGMT (Fig. 3I,J). Bioluminescence images revealed that no significant differences in the tumors volume and the levels of MGMT protein between P-GBM2 cells transduced with BACH1 and those cells transduced with vector control in the absence of p53 (Fig. 3K). And mice bearing tumors derived from P-GBM2 cells stable expressing BACH1 plus shp53 had no significant difference in the survival time compared with mice bearing tumors derived from P-GBM2 cells stable expressing empty vector plus shp53 in response to TMZ (Fig. 3L). Taken together, these results indicate that p53 knockdown abolished the effects of BACH1 on TMZ resistance *in vitro* and *in vivo*.

### BACH1 impairs the association between p53 and SP1 via competitive binding p53

**Figure 4:**
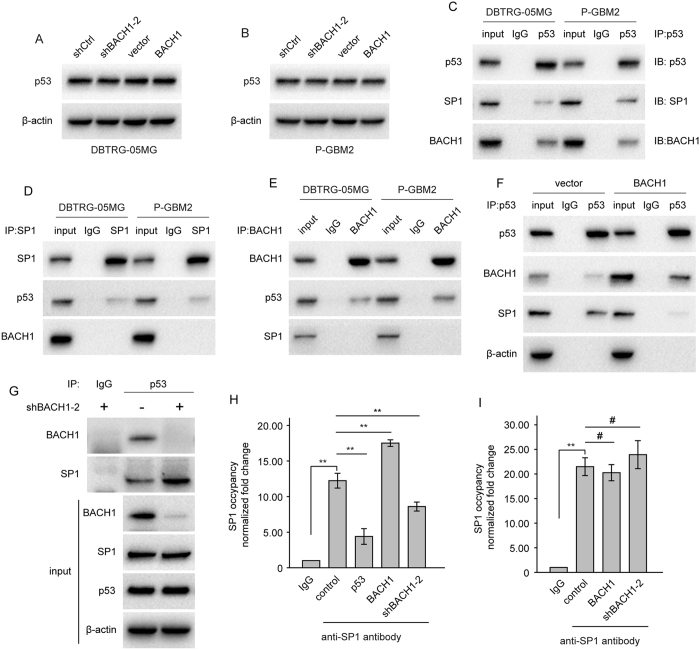
BACH1 affects the expression of MGMT through impairing the association between p53 and SP1. (**A**) Western blot analysis of p53 expression in DBTRG-05MG cells transfected with BACH1 expression plasmid or shBACH1-2. (**B**) Western blot analysis of p53 expression in P-GBM2 cells transfected with BACH1 expression plasmid or shBACH1-2. (**C**–**E**) Lysates from GBM cells were incubated in the presence of anti-IgG, or anti-p53, or anti-SP1, or anti-BACH1 antibody. Immunoprecipitated material was subjected to SDS-PAGE, and Westernblot analysis was performed with anti-p53, anti-SP1 and anti-BACH1 antibodies. (**F**,**G**) Co-IP was performed using lysates prepared from DBTRG-05MG cells transfected with BACH1, or vector control, or shBACH1-2, or shCtrl using anti-IgG or anti-p53 antibody. And Westernblot analysis was performed with indicated antibodies. (**H**) DBTRG-05MG cells were separately transfected with wild-type p53, shBAHC1, or BACH1. ChIP assays were performed using anti-IgG or anti-SP1 antibody. The eluted DNA was subjected to qRT-PCR with the specific primer set for the MGMT promoter region. (**I**) DBTRG-05MG cells stably expressing shp53 were separately transfected with shBAHC1 or BACH1. ChIP assays were performed using anti-IgG or anti-SP1 antibody. The eluted DNA was subjected to qRT-PCR with the specific primer set for the MGMT promoter region. Student’s t-tests and One-way ANOVA were performed. Data are presented as mean ± SEM (**P < 0.01, ^#^*P* > 0.05).

To further explore the relationship between BACH1 and p53, GBM cells upregulating or downregulating BACH1 were employed. Western blot revealed that the expression of p53 was not affected by BACH1 overexpression or downregulation (Fig. 4A,B). In mouse, BACH1 forms a complex with p53, resulting in negative regulation of oxidative stress-induced cellular senescence^27^,^28^. And p53 mediated down-regulation of MGMT via interaction with SP1^22^. Co-immunoprecipitate was used to explore the interaction among BACH1, p53 and SP1. Endogenous BACH1 and SP1 could be co-immunoprecipitated with anti-p53 antibody (Fig. 4C). However BACH1 or SP1 could not be co-immunoprecipitated with anti-SP1 antibody or anti-BACH1 antibody (Fig. 4D,E). Furthermore, BACH1 overexpression significantly decreased the interaction between p53 and SP1, and depletion of BACH1 increased SP1-p53 binding (Fig. 4F). Moreover, the binding of SP1 to the MGMT promoter region was significantly increased in BACH1 overexpressing cells and decreased in BACH1-depleted cells (Fig. 4G). In addition, SP1 occupancy at the MGMT promoter was unaffected by BACH1 in cells p53 knockdown (Fig. 4H). These results suggest that BACH1 affects the expression of MGMT through blocking the association between p53 and SP1 via competitive binding p53.

### Low expression of BACH1 plus wild-type p53 correlate with better prognosis in GBM patients receiving TMZ therapy

**Figure 5:**
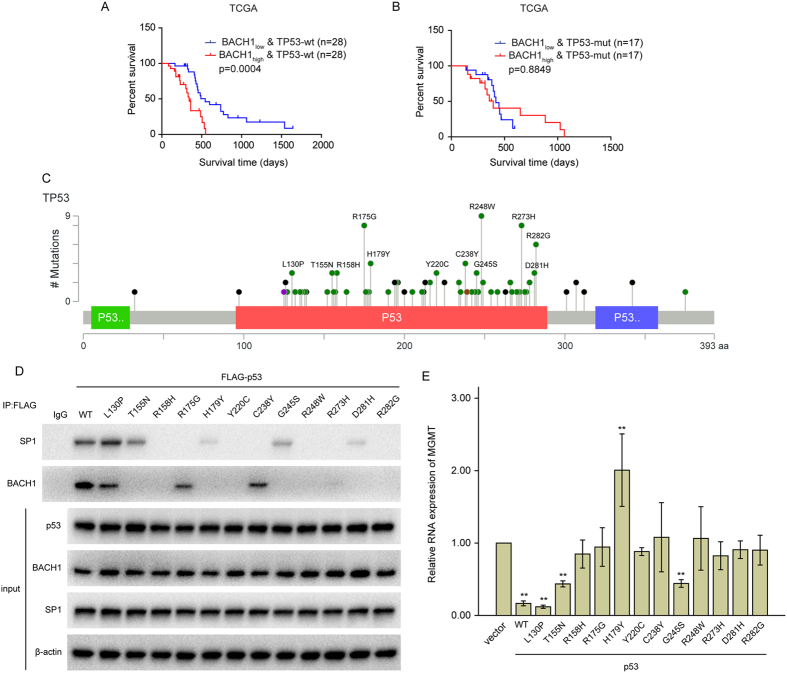
Low expression of BACH1 plus wild-type p53 correlate with better prognosis in GBM patients receiving TMZ therapy. (**A**,**B**) Kaplan–Meier curves showing the overall survival of GBM patients with high or low expression of BACH1 & TP53-wt(A) or TP53-mut(B) in 90 GBM patients receiving TMZ therapy using the TCGA database. (**C**) the hotspot mutation sites of TP53 in GBM. L: Leucine; P: Proline; T: Threonine; N: Asparagine; R: Arginine; H: Histidine; G: Glycine; Y: Tyrosine; C: Cystine; S: Serine; W: Tryptophan; D: Asparticacid. (**D**) DBTRG-05MG cells were separately transfected with FLAG-p53-WT or FLAG-p53-mutants. Co-IP assays were performed using anti-IgG or anti-FLAG antibody. (**E**) The RNA level of MGMT in DBTRG-05MG cells transfected with FLAG-p53-WT or FLAG-p53-mutants. Student’s t-tests were performed. Data are presented as mean ± SEM (**P < 0.01).

To validate the association between BACH1 and p53 in GBM patients, data from 90 GBM patients receiving TMZ therapy in the TCGA database that contained integrated survival information were selected for Kaplan–Meier survival curve analysis. As shown in Fig. 5A, GBM patients with lower than the median level of BACH1 expression & wild-type p53 had much better overall survival than those with high BACH1 expression levels & wild-type p53. However, there was no significant difference in the overall survival of patients with TMZ therapy in the BACH1 low expression & p53-mutant and BACH1 high expression & p53-mutant groups (Fig. 5B).

Previous experiments have shown that wild-type p53 protein binds to SP1^22^. It was of some interest, therefore, to determine whether p53 mutants failed to bind to SP1, an extensive series of hotspot mutation sites in GBM were created. These mutants are listed in Fig. 5C with their codon number (1–393 codons), the amino acid present in wild-type p53, and the alteration made in specific mutants. Co-immunoprecipitation was employed to demonstrate p53-SP1 and p53-BACH1 interactions. Each FLAG-p53-mutant was transfected into DBTRG-05MG cells for 48 h. The cell lysates were then immunoprecipitated with a FLAG-specific monoclonal antibody. The results revealed that SP1 could be co-immunoprecipitated in cells transfected with p53-WT, p53-mutant-L130P/T155N/H179Y/G245S/D281H (Fig. 5D). And BACH1 could be co-immunoprecipitated in cells transfected with p53-WT, p53-mutant-L130P/ R175G/C238Y (Fig. 5D). The RNA level of MGMT obviously decreased in cells transfected with p53-mutant-L130P/T155N/G245S, and increased in cells transfected with p53-mutant-H179Y (Fig. 5E). Together, these findings shed light on the clinical significance of these proteins in the regulation of TMZ resistance.

## Discussion

In this study, our *in vitro* and *in vivo* experiments, complemented by analysis of clinical samples, demonstrated that BACH1 may be a novel key molecule that is responsible for TMZ resistance in wild-type p53 GBMs. We initially detected that BACH1 was upregulated in GBM, and GBM patients with low levels of BACH1 would benefit from TMZ-based therapy. Moreover, ectopic expression of BACH1 in GBM cells promoted acquired resistance to TMZ, whereas depletion of BACH1 sensitized TMZ-refractory cells to TMZ *in vitro* and *in vivo*. These data are further supported by the findings that xenografts expressing low level of BACH1 exhibited significantly better survival when treated with TMZ compared with xenografts expressing high level of BACH1. Thus, our findings shed insight on the importance of BACH1 in TMZ resistance and the applicability of BACH1 as a potential prognostic and predictive marker of response to TMZ.

As a heme-binding protein, BACH1 forms heterodimer through the BTB-POZ domain which binds to the DNA-binding complex and is involved in the regulation of the chromosomal structure, which turns out the transcriptional repression or activation^17^. Overexpression of BACH1 promoted MGMT expression at the transcriptional level via the activity of the MGMT distal promoter in cells with MGMT promoter hypomethylation, but not in cells with MGMT promoter methylation. However, BACH1 could not occupy the promoter of MGMT. The MGMT promoter, like that of many housekeeping genes, does not have any TATA or CAAT boxes, but contain six putative SP1-binding sequences and two AP-1 binding sequences^20^,^21^,^29^,^30^,^31^. BACH1 overexpression enhanced SP1 binding to its consensus sequence on the MGMT promoter and promoted transcription through promoter activity. Further research indicated that BACH1 did not affect the MGMT expression in U138 cells which has a nonfunctional p53^23^,^24^. In addition, knockdown of p53 abolished the effects of BACH1 on TMZ resistance and the enhancement effect of BACH1 on MGMT. P53 serves as a transcription factor, and has a wide range of functions in cell cycle regulation, cell cycle arrest, DNA repair and apoptosis through transactivation of specific genes in response to DNA damage (ionizing radiation, IR and UV light, chemotherapeutic agents) and other cellular stress signals^32^,^33^. Previously it was reported that p53 has an impact on the sensitivity of glioma cells to TMZ, ACNU and BCNU^9^,^34^,^35^. Interestingly, in spite of the lack of p53-binding sites in the MGMT promoter, several studies have explored the impact of p53 on MGMT expression^36^,^37^,^38^. Bocangel *et al*. reported that wild-type p53 sequesters SP1 to prevent its binding to the cognate *cis* elements in the MGMT promoter and thus inhibits MGMT expression^22^. Our study confirmed a stable interaction of p53 with SP1 in GBM cells. And p53 overexpression prevented SP1 from binding to the MGMT promoter and repressed MGMT transcription. Previous studies have reported that BACH1 forms a complex with p53, resulting in negative regulation of oxidative stress-induced cellular senescence^27^,^28^. We revealed a direct physical interaction between BACH1 and p53 in GBM cells. And BACH1 overexpression significantly decreased the interaction between p53 and SP1. In addition, the binding of SP1 to the MGMT promoter region was significantly increased in BACH1 overexpressing cells and decreased in BACH1-depleted cells. However, SP1 occupancy at the MGMT promoter was unaffected by BACH1 in cells p53 knockdown. Thus, our findings unveiled that BACH1 dictates MGMT expression and TMZ resistance via prevent the interaction between SP1and p53.

As a master regulator of cell apoptosis, p53-dependent apoptosis also contributes to chemotherapy induced cell death by transcriptionally activating pro-apoptotic Bcl-2 family members (e.g., Bax, Bak, PUMA, and Noxa) and repressing anti-apoptotic Bcl-2 proteins (Bcl-2, Bcl-XL) and IAPs (survivin)^39^,^40^,^41^,^42^. Our study only focuses on p53 as a regulator of MGMT. Therefore, does BACH1 impact the pro-apoptosis pathways of p53? It will be interesting to explore this possibility in the future.

Alteration of tumor suppressor p53 is seen in approximately 25–30% of primary GBM, making it the most common molecular abnormality in glioma^43^. Certain frequent TP53 mutations lead to the loss of function of tumor suppressor p53. Analysis of BACH1 expression and p53 status using the TCGA database revealed that GBM patients with BACH1 low expression & wild-type p53 had much better overall survival than those with high BACH1 expression levels & wild-type p53 in response to TMZ. Furthermore, the RNA level of MGMT markedly decreased in cells transfected with p53-mutant-L130P/T155N/G245S, and increased in cells transfected with p53-mutant-H179Y. Ectopic expression of wild-type p53 significantly reduced MGMT promoter activity through sequestering SP1^22^. In contrast, ectopic expression of p53-mutant-H179Y which also binds to SP1 enhanced the expression of MGMT. Although we have revealed that BACH1 controls MGMT expression via wild-type p53, the roles of TP53 mutation in MGMT promoter activity still remain elusive. Future studies are needed to address the active roles of TP53 mutation in development of chemoresistance.

Collectively, we showed that BACH1 is critical in modulating TMZ resistance in GBMs through removing the RNA transcriptional silencing function of p53 at the MGMT gene. Our study not only reveals a novel mechanism underlying acquired TMZ resistance in wild-type p53 GBMs but also has important implications in the development of treatment strategies for TMZ-resistant GBMs.

## Materials and Methods

### Cell lines and patient tissue specimens

Gene expression data from the TCGA were downloaded from the TCGA database (http://tcga-data.nci.nih.gov). Cell lines and patient samples information are detailed in the Supplementary Materials. This study was approved by the institutional review board and the Research Ethics Committee of Nanjing Medical University (Nanjing, Jiangsu, China). Informed consents were obtained from all participants. All methods were performed in accordance with the approved guidelines.

### Plasmids construction, transfection and stable cell establishment

See Supplementary Materials for details on plasmids construction, transfection, and stable cell establishment.

### Quantitative real-time PCR

Real-time PCR was performed according to the manufacturer’s instructions. The primers for has-MGMT: forward primer, 5′-ACCGTTTGCGACTTGGTACTT-3′; reverse primer, 5′-GGAGCTTTATTTCGTGCAGACC-3′. Has-β-actin mRNA was also amplified in the same PCR reactions as an internal control using the primers: forward primer, 5′-CATGTACGTTGCTATCCAGGC-3′; reverse primer, 5′-CTCCTTAATGTCACGCACGAT-3′. All experiments were performed using biological triplicates and experimental duplicates. The relative expression was calculated via the 2^−ΔΔCt^ method.

### Western blotting

Western blotting was performed as described previously^44^, and images were captured by Bio-Rad ChemiDoc XRS+ (Bio-Rad, CA, USA). The antibodies used in this study were: MGMT, γ-H2AX, H2AX, BACH1 (Abcam, USA); Caspase-3, p53, SP1 (Cell Signaling Technology, USA); FLAG (Sigma-Aldrich). The antibody against β-actin (Santa Cruz, USA) was used as a control.

### Colony formation assay

Cells stably expressing BACH1, shBACH1 or shCtrl were independently plated onto 60-mm dishs (1000 in a 60-mm dish) followed by being treated with TMZ (200 μM) for 48 hours, and then the culture media were refreshed without TMZ. After 10 to 12 days, visible colonies were fixed with 100% methanol and stained with 0.1% crystal violet in 20% methanol for 15 minutes. Colony-forming efficiency was calculated as the number of colonies/plated cells ×100%.

### Annexin V-FITC/PI staining

The number of apoptotic cells was counted using an AnnexinV-FITC/PI Apoptosis Detection Kit (BD Biosciences, CA, USA) according to the manufacturer’s protocol. Apoptotic cells were analyzed on a Gallios Flow cytometer (Beckman, CA, USA). The results are presented as the percentage of apoptotic cells relative to the total number of cells.

### Co-immunoprecipitation (Co-IP)

Co-immunoprecipitation assays were performed using the Co-immunoprecipitation kit (Thermo Scientific, MA, USA). GBM cells were lysed in SDS lysis buffer on ice for 30 min, and the supernatants were collected by centrifugation at 4 °C and 14,000 × g for 15 min. The cleared protein lysates were then incubated with anti-FLAG antibody, anti-p53 antibody, anti-SP1 antibody or BACH1 antibody with rotation overnight at 4 °C. Then, the mixtures were incubated with immobilized protein A/G beads (Thermo Scientific) with rotation at 4 °C for 2 h. The beads were collected by centrifugation at 3000 × g for 2 min, and then washed five times with 0.5 ml IP wash buffer. SDS loading buffer was added to the beads, and the samples were denatured at 95 °C for 8–10 min. Finally, the supernatants were collected and stored at −80 °C or immediately analyzed by WB.

### Chromatin immunoprecipitation

Chromatin immunoprecipitation (ChIP) assays were performed according to the manufacturer’s protocol (the EZ-magna ChIP kit, Millipore, Bedford, MA, USA). Details are described in the Supplementary Methods.

### Methylation-specific PCR assay

The methylation status of the MGMT promoter were evaluated by methylation-specific PCR (MSP) assay as described previously^45^,^46^. Genomic DNA was extracted from the cells using a QIAamp DNA Mini Kit (Qiagen). Sodium bisulfite conversion of 200 ng of the purified DNA was performed using an EpiTect Bisulfite Kit (Qiagen) according to the manufacturer’s protocol. MSP of bisulfate converted DNA was carried out in a nested, two-stage PCR approach using GeneAmp PCR System 2700 (Applied Biosystems, Grand Island, NY). Amplified PCR products were separated by 3% agarose gel electrophoresis and visualized with ethidium bromide.

### MethyLight assay

Genomic DNA was extracted from the cells using a QIAamp DNA Mini Kit and subjected to bisulfite conversion using an EpiTect Bisulfite Kit followed by quantitative real-time PCR of bisulfite-converted DNA using SYBR Green master mix (Roche Applied Science, Upper Bavaria, Germany) with primers and probe specific to methylated fraction of the MGMT promoter. Probe and forward primer sequences: probe, 6FAM-CCTTACCTCTAAATACCAACCCCAAACCCG-BHQ-1; forward primer, 5′-CTAACGTATAACGAAAATCGTAACAACC-3′; reverse primer, 5′-AGTATGGAAGGGTAGGAAGAATTCG-3′. Alu was utilized as a calibrator with following probe and primers: probe, 6FAM-CCTACCTTAACCTCCC-BHQ-1; forward primer, 5′-GGTTAGGTATAGTGGTTTATATTTGTAATTTTAGTA-3′; reverse primer, 5′-ATTAACTAAACTAATCTTAAACTCCTAACCTCA-3′.

### Orthotopic xenograft studies

Animal experiments were approved by the Animal Management Rule of the Chinese Ministry of Health (documentation 55, 2001) and were in accordance with the approved guidelines and the experimental protocol of Nanjing Medical University (Nanjing, China). All experiments involving mice were provided by The Model Animal Research Center of Nanjing University (Nanjing, China). For orthotopic xenograft studies, GBM cells (2.5 × 10^5^) stably expressing BACH1, shBACH1–2 or shCtrl were injected intracranially into the striatum of NOD/SCID mice using a stereotactic device (coordinates: 2 mm anterior, 2 mm lateral, 3 mm depth from the dura). At 1 week, the tumor-bearing mice were given TMZ or placebo by oral gavage (66 mg/kg daily for 5 days per week for three cycles) and sacrificed upon signs of tumor formation (rough coat, hunching, and weight loss). Tumors were measured by luminescence imaging (IVIS Spectrum, PerkinElmer, USA) each week.

### Immunohistochemistry (IHC)

Immunohistochemistry to detect MGMT, γ-H2AX and cleaved caspase-3(Cell Signaling Technology) in nude mouse xenograft tumor tissues was performed as described previously^47^.

### Statistical analysis

Quantitative data are presented as means ± SEM. The statistical significance levels were set at P < 0.05(*) and P < 0.01(**). One-way ANOVA and Student’s t-tests were used to perform comparisons. Biostatistical analysis was performed using Office SPSS software (SPSS version 17.0) and GraphPad software (GraphPad Prism 5). All statistical tests were two-sided.

## Additional Information

**How to cite this article:** Nie, E. *et al*. BACH1 Promotes Temozolomide Resistance in Glioblastoma through Antagonizing the Function of p53. *Sci. Rep.* **6**, 39743; doi: 10.1038/srep39743 (2016).

**Publisher's note:** Springer Nature remains neutral with regard to jurisdictional claims in published maps and institutional affiliations.

## Supplementary Material

Supplementary Materials and Data
