# Supplementary material for: BACH1 Promotes Temozolomide Resistance in Glioblastoma through Antagonizing the Function of p53
Source: Sci Rep. 2016 Dec 21;6:39743. doi: 10.1038/srep39743 (PMC5175153; doi:10.1038/srep39743)
Supplement: Supplementary Materials and Data [file srep39743-s1.pdf]

BACH1 Promotes Temozolomide Resistance in Glioblastoma through Antagonizing the Function of p53

Er Nie[1], Xin Jin[1], Weining Wu[1], Tianfu Yu[1], Xu Zhou[1], Tongle Zhi[1], Zhumei Shi[1,2], Junxia Zhang[1], Ning Liu[1,3], and Yongping You[1,3,*]

[1]Department of Neurosurgery, the First Affiliated Hospital of Nanjing Medical University, Nanjing 210029, China.

[2]State Key lab of Reproductive Medicine, Department of Pathology, Collaborative Innovation Center for Cancer Personalized Medicine, Cancer Center, Nanjing Medical University, Nanjing 210029, China;

[3]Chinese Glioma Cooperative Group (CGCG)

*To whom correspondence should be addressed. Tel: +86 025 83718836; Fax: +86 025 83718836; Email: yypl9@njmu.edu.cn

Supplementary Materials and Methods

Patients and samples

A total of 26 patients (17 males and 9 females) with a median age of 49.6 years (range, 21–67years) who had been treated surgically at the Department of Neurosurgery, The First Affiliated Hospital of Nanjing Medical University (Nanjing, China), between January 2012 and October 2013 were selected for the study. Among the 26 cases, 21 tumor samples and five non-tumorous brain specimens were obtained for analysis from the patients registered at the hospital. Informed consent was obtained from each subject, and the study was approved by the ethics committee of the hospital.

The glioma tissue samples were histologically diagnosed by the Department of Pathology at The First Affiliated Hospital of Nanjing Medical University according to the WHO classification[1]. As controls, five human non-tumor brain tissue (NBT) samples were obtained primarily from the cortex of patients with decompressive surgery after physical injury to the brain. Samples were immediately snap-frozen in liquid nitrogen until use. All methods were performed in accordance with the approved guidelines.

Cell culture and reagents

The human glioblastoma multiforme (GBM) cell lines A172, U87, U251, LN229, U138 and T98 were purchased from Shanghai Cell Bank of the Chinese Academy of Sciences (Shanghai, China). DBTRG-05MG cell line was bought from Sigma-Aldrich (St. Louis, MO, USA). Normal human astrocytes (NHAs) were obtained from Lonza (Walkersville, MD, USA) and cultured in the provided astrocyte growth media supplemented with rhEGF, insulin, ascorbic acid, GA-1000, L-glutamine and 5% FBS. The primary GBM1 cell line was established in July 2013 from cells taken from a

patient with right frontal glioblastoma, and the primary GBM2 cell line was established in October 2013 from cells taken from a patient with right tempus glioblastoma. Tumor tissue was collected from the operating room during resection with written patient consent, under The First Affiliated Hospital of Nanjing Medical University Institutional Review Board approved protocols. Tissue was obtained from regions of viable tumor. Within 2 hours, the samples were either finely minced prior to organoid formation or were dissociated into single-cell suspensions, red blood cells removed by brief hypotonic lysis, and counted for cell number and viability using trypan blue. Primary cell lines were maintained in primary serum-free cultures grown on laminin. All GBM cell lines were validated in October 2014 by short tandem repeat DNA fingerprinting using the AmpFlSTR Identifiler kit according to the manufacturer's instructions (Applied Biosystems, CA, USA). All GBM cell lines were preserved in liquid nitrogen to maintain authenticity. The cells used for the experiments were replenished from frozen stocks every 3 months. Cells were cultured in 5% CO_2 at 37 °C in Dulbecco's modified Eagle's medium (DMEM, Gibco, CA, USA) supplemented with 10% fetal bovine serum (Gibco).

Plasmids construction, transfection and stable cell establishment

The entire coding sequence of BACH1 and p53 was obtained from HUVEC mRNA by RT-PCR. BACH1 and p53 cDNA were purified by Genechem (Shanghai, China) and were cloned into pcDNA3.1-FLAG vector to generate pcDNA3.1-FLAG-BACH1 and pcDNA3.1-FLAG-p53 recombinant plasmid. Recombinant plasmids expressing FLAG-p53-mutants were provided by Genechem (Shanghai, China). Transient transfection was performed using Lipofectamine™ 2000 transfection reagent (Invitrogen) according to the manufacturer's instructions. For stable transfection,

GBM cells were transfected with BACH1, shBACH1, or shp53 (Genechem) lentiviral particles according to the manufacturer's protocol. Scrambled lentiviral particles were used as a control. After 48 h of incubation, the medium was replaced with DMEM containing 5μg/ml puromycin. After maintenance for 3-4 weeks in selection media, puromycin-resistant colonies were selected and screened for BACH1 or p53 expression.

Chromatin immunoprecipitation

Chromatin immunoprecipitation (ChIP) assays were performed using the EZ-magna ChIP kit (Millipore, Bedford, MA, USA) according to the manufacturer's protocol. The chromatin solution was immunoprecipitated overnight at 4°C with 50 μl of protein A/G magnetic beads (Millipore) and 5 μg of anti-FLAG antibody, anti-SP1 antibody, anti-p65 antibody, anti-C-JUN antibody or p-300 antibody (Abcam). Control samples were immunoprecipitated with 5 μg IgG (Millipore). After immunoprecipitation, the beads were washed sequentially with low-salt buffer, high-salt buffer, LiCl buffer, and TE buffer each for 5 min at 4˚C. The immunoprecipitated DNA was then eluted by incubation in 100 μl of elution buffer (0.1M NaHCO3 and 1% SDS) containing 10 μg proteinase K (Millipore) at 62°C for 2 h with rotation. The eluted DNA was purified using the columns and buffers contained in the kit (Millipore), and was finally re-dissolved in 50 μl of PCR-grade water. The eluted DNA was subjected to quantitative PCR using SYBR Green master mix (Roche Applied Science, Upper Bavaria, Germany).

List of primers used for CHIP

	Forward primer	Reverse primer	Remarks
MGMT promoter region 1	CCTCTTAGGCTTCTG GTGGC	TGGGGTTGTGTGGAC GTTA	(-926~-697)

MGMT promoter region 2	TCTGGCAGTGTCTAG GCCAT	CGCCCGCTTAGTGAG AATCC	(-647~-440)
MGMT promoter region 3	ACAGGAAAAGGTAC GGGCCAT	GCCCTTCGGCCGGTA CAA	(-359~-267)
MGMT promoter region 4	ATGCGCAGACTGCCT CAG	CACTCACCAAGTCGC AAACG	(-195~+120)
MGMT promoter for SP1	GCCCCGGCCCCGCCC CCGCGCG	GCTATGCGTTATTGA GCACGCG	2
MGMT promoter for p65	ATGCGCAGACTGCCT CAG	CCGAGGACCTGAGA AAAGCA	3
MGMT promoter for C-JUN	GCTCCAGGGAAGAG TGTCCTCTGCTCCCT	GGCCTGTGGTGGGC GATGCCGTCCAG	4
MGMT promoter for p-300	GACAGGAAAAGGTA CGGGCCATT	GAGCCGAGGACCTG AGAAAAGCAAG	5

Supplementary Figures

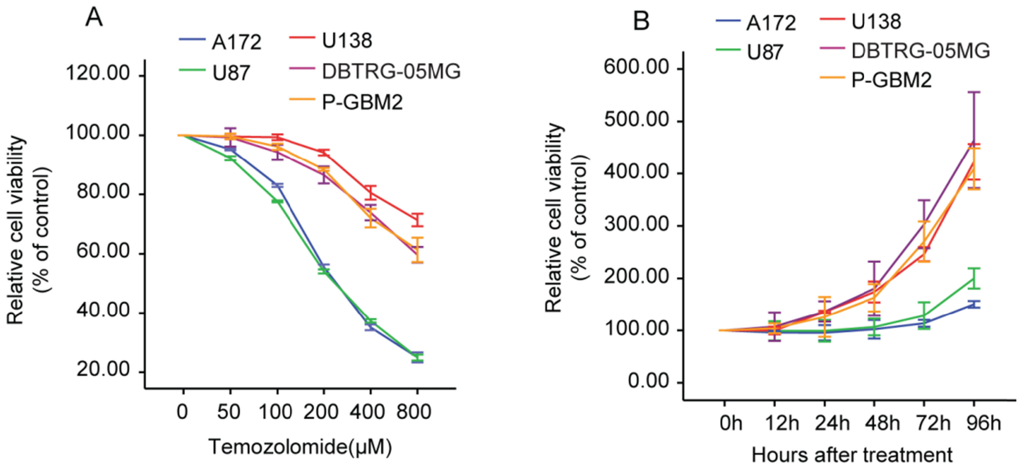

Supplementary Figure 1.

A, Cell proliferation was evaluated in glioblastoma cells with or without the temozolomide treatments at different doses. CCK8 assay was performed 48 h after treatment. B, The cell proliferation in 200 μM temozolomide treatments were tested at indicated time using CCK8 assay. One-way ANOVA were performed. Data are

presented as mean ± SEM.

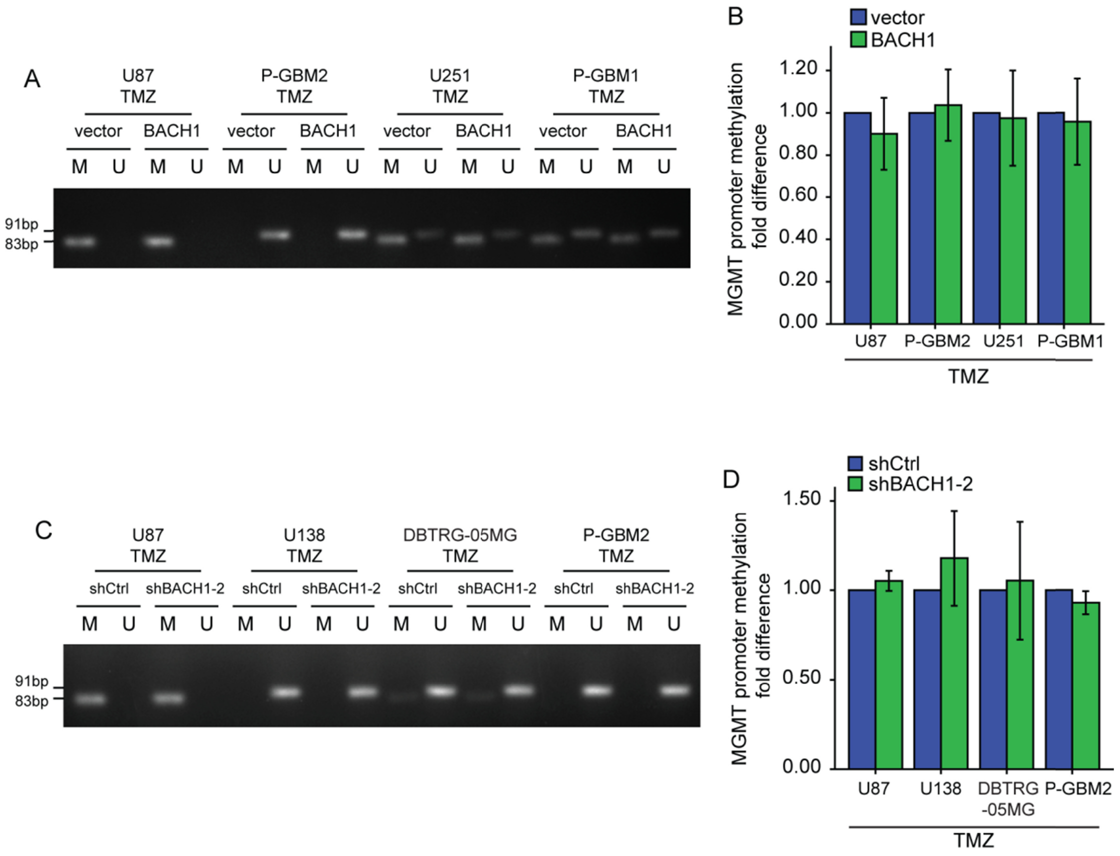

Supplementary Figure 2.

A and C, the methylation status of the MGMT promoter in GBM cells transfected with BACH1 expression construct or shBACH1 in the presence of temozolomide(200 μM) was observed by MSP assay. PCR products in the M lanes and U lanes indicate methylated and unmethylated status of the MGMT promoter, respectively. B and D, The methylation status of the MGMT promoter was examined with MethyLight assay in GBM cells treated with BACH1 expression construct or shBACH1 in the presence of temozolomide(200 μM). qPCR of bisulfite-converted DNA with the primers and probes specific to the methylated fraction of the MGMT promoter. Student's t-tests were performed. Data are presented as mean ± SEM.

References

1 Louis, D. N. *et al.* The 2007 WHO classification of tumours of the central nervous system. *Acta Neuropathol* **114**, 97-109, doi:10.1007/s00401-007-0243-4 (2007).

2 Bocangel, D., Sengupta, S., Mitra, S. & Bhakat, K. K. p53-Mediated down-regulation of the human DNA repair gene O6-methylguanine-DNA methyltransferase (MGMT) via interaction with Sp1 transcription factor. *Anticancer research* **29**, 3741-3750 (2009).

3 Lavon, I. *et al.* Novel mechanism whereby nuclear factor kappaB mediates DNA damage repair through regulation of O(6)-methylguanine-DNA-methyltransferase. *Cancer Res* **67**, 8952-8959, doi:10.1158/0008-5472.can-06-3820 (2007).

4 Bhakat, K. K. & Mitra, S. Regulation of the human O(6)-methylguanine-DNA methyltransferase gene by transcriptional coactivators cAMP response element-binding protein-binding protein and p300. *J Biol Chem* **275**, 34197-34204, doi:10.1074/jbc.M005447200 (2000).

5 Kitange, G. J. *et al.* Retinoblastoma Binding Protein 4 Modulates Temozolomide Sensitivity in Glioblastoma by Regulating DNA Repair Proteins. *Cell reports* **14**, 2587-2598, doi:10.1016/j.celrep.2016.02.045 (2016).
